# A Comparative Study on the Effect of Exosomes Secreted by Mesenchymal Stem Cells Derived from Adipose and Bone Marrow Tissues in the Treatment of Osteoarthritis-Induced Mouse Model

**DOI:** 10.1155/2021/9688138

**Published:** 2021-09-27

**Authors:** Hoda Fazaeli, Naser Kalhor, Leila Naserpour, Faezeh Davoodi, Mohsen Sheykhhasan, Seyed Kamal Eshagh Hosseini, Mohammad Rabiei, Azar Sheikholeslami

**Affiliations:** ^1^Department of Mesenchymal Stem Cells, Academic Center for Education, Culture, and Research (ACECR), Qom Branch, Qom, Iran; ^2^Department of Reproductive Biology, Academic Center for Education, Culture, and Research (ACECR), Qom Branch, Qom, Iran; ^3^Pediatric Hematology Research Center, Faculty of Medicine, Qom University of Medical Sciences, Qom, Iran; ^4^Department of Biology, Faculty of Science, Azad Islamic University of Qom, Qom, Iran

## Abstract

**Background:**

Exosomes as extracellular vesicles (EVs) are nanoscale intercellular messengers secreted from cells to deliver biological signals. Today, exosomes have become a new field of research in regenerative medicine and are considered as potential therapies to control inflammation and wound healing and enhance and improve healing in many diseases. Given the global burden of osteoarthritis (OA) as the fastest-growing health condition and one of the major causes of physical disability in the aging population, research to establish EVs as therapeutic products can meet the basic clinical needs in the management of osteoarthritis and provide a therapeutic solution.

**Objectives:**

The present study is aimed at evaluating the regenerative potentials of the exosomes secreted from adipose and bone marrow tissue-derived mesenchymal stem cells (AD- and BM-MSCs) in ameliorating the symptoms of OA.

**Method:**

In this experimental study, AD- and BM-MSCs were isolated and cultured in the laboratory until passage 3. Finally, these cells' secreted exosomes were isolated from their conditioned medium. Ciprofloxacin-induced OA mouse models underwent intra-articular injection of exosomes from AD-MSCs and BM-MSCs. Finally, the expression levels of collagen I and II, sox9, and aggrecan genes using real-time PCR, histological analysis, and immunohistochemical (IHC) studies were performed.

**Results:**

Real-time PCR data showed that although the expression level of collagen type II was lower in both exosome-treated groups than the normal, but it was significantly increased in comparison with the sham and OA, with higher expression in BM-Exo rather than AD-Exo group. Similarly, the histological staining and IHC results have provided almost identical data, emphasizing on better therapeutic effect of BM-MSCs-exosome than AD-MSCs-exosome.

**Conclusion:**

BM-MSCs secreted exosomes in comparison with AD-MSCs could be considered as a better therapeutic option to improve osteoarthritis and exhibit potential as a disease-modifying osteoarthritis cell-free product.

## 1. Introduction

As a degenerative and inflammatory joint condition [1], osteoarthritis (OA) affects 10% of men and 18% of women over the age of 60 and consequently leads to cause a significant healthcare burden on society [2]. The OA symptoms are subchondral bone sclerosis; synovial inflammation includes synovial hyperplasia, fibrosis, and thickening of the synovial capsule, activated synoviocytes, and in some cases, lymphocytic infiltrate (B- and T-cells as well as plasma cells), cartilage degradation, ligament calcification, and osteophyte formation [3]. The pathogenesis of OA involves the interaction between mechanical, genetic, metabolic, and inflammatory mechanisms [4, 5]. Traditional pharmacological therapies and nonpharmacological treatments such as surgical procedures can only provide symptomatic relief; however, damaged cartilage cannot be restored effectively [6]. The treatment of OA has always been difficult due to the lack of articular blood supply and the fact that chondrocytes are highly differentiated cells with low proliferative and migratory capacity [7].

With advances in regenerative medicine, mesenchymal stem cells (MSCs) have emerged as an alternative cellular therapy for the treatment of OA. MSCs are a form of multipotent stromal cells that can differentiate into various cells, including osteocytes and chondrocytes, and are widely used in cartilage regenerative therapies [8, 9]. Advances in stem cell transplantation therapy have shown promise in treating OA. MSCs, such as bone marrow-derived MSCs (BM-MSCs) [10, 11] and adipose-derived MSCs (AD-MSCs) [12, 13], have been commonly used for the treatment of OA in the last decade. According to current research, MSCs have the following functions: interacting with the immune system to promote immuno-regulation [14], migrating to the injury site to improve peripheral tissue tolerance, preventing the inflammatory factor release, promoting tissue repair, and increasing the activity of injured cells [15, 16], as well as high potential for multidirectional differentiation and reproduction [16].

Many drawbacks of stem cell transplantation therapy, such as the possibility of tumor development, ethical issues, and graft rejection, have yet to be overcome [17].

Exosomes as extracellular vesicles (EVs) secreted from MSCs are a mixed population of heterogeneous membranous vesicles with a diameter of 50 to 150 nm, contain diverse ingredients that serve as essential intercellular communication mediators [18, 19]. Nucleic acids (mRNAs and microRNAs), proteins, and bioactive lipids can all be transported by exosomes [20, 21]. Also, exosomes contain some cytokines, including transforming growth factor-1, hepatocyte growth factor, fibroblast growth factor, and vascular endothelial growth factor, which have anti-inflammatory, antiapoptosis, antifibrosis, proangiogenesis, promitosis, and prowound healing effects [22, 23] which make them potent to cause biological responses in the cells they are delivered to. These vesicles form a vesicle-mediated transport system that controls a variety of physiologic and pathological activities [24].

Recent research has bolstered the importance of MSC-derived exosomes in the regulation of cell migration, proliferation, differentiation, and extracellular matrix formation [25]. Furthermore, studies have shown that MSCs' exosomes aid cartilage repair and regeneration by modulating immunological reactivity, decreasing apoptosis, and promoting proliferation [26, 27].

Recently, numerous investigations have been carried out to evaluate the potential role of secreted exosomes from different sources of derived stem cells.

Adipose and bone marrow tissues are two of the most extensively studied MSCs' sources used in regenerative medicine [28–30]. Although in comparison to AD-MSCs which can be easily isolated from adipose tissues [31, 32], the clinical application of BM-MSCs has been limited due to the painful and invasive bone marrow aspiration procedure [33]; it has been demonstrated that they have greater osteogenic and chondrogenic differentiation ability [34].

Since secreted exosomes from AD-MSCs and BM-MSCs have not been compared concerning their efficacy in the treatment of OA, it remains unclear whether both exhibit equal regenerative capabilities. So, the present study is aimed at evaluating the regenerative potentials of the exosomes from two sources of MSCs in ameliorating the symptoms of OA.

## 2. Materials and Methods

### 2.1. Ethics Statement and Study Design

This interventional experimental study was approved by the ethics committee of Qom Azad University (IR.IAU.QOM.REC.1398.023). In this study, we compared the effect of exosomes derived from MSCs from two sources of bone marrow and adipose tissues on the OA mouse model, and the study was defined in 5 groups as follows:

1. *Healthy Control Group (Normal)*. Healthy BALB/c mice with no treatment
2. *OA Control Group (OA)*. OA-induced BALB/c mice with no treatment
3. *Sham Group*. OA-induced BALB/c mice treated with phosphate-buffered saline (PBS)
4. *BM-Exo Group*. OA-induced BALB/c mice treated with exosomes derived from BM-MSCs
5. *AD-Exo Group*. OA-induced BALB/c mice treated with exosomes derived from AD-MSCs

**Figure 1:**
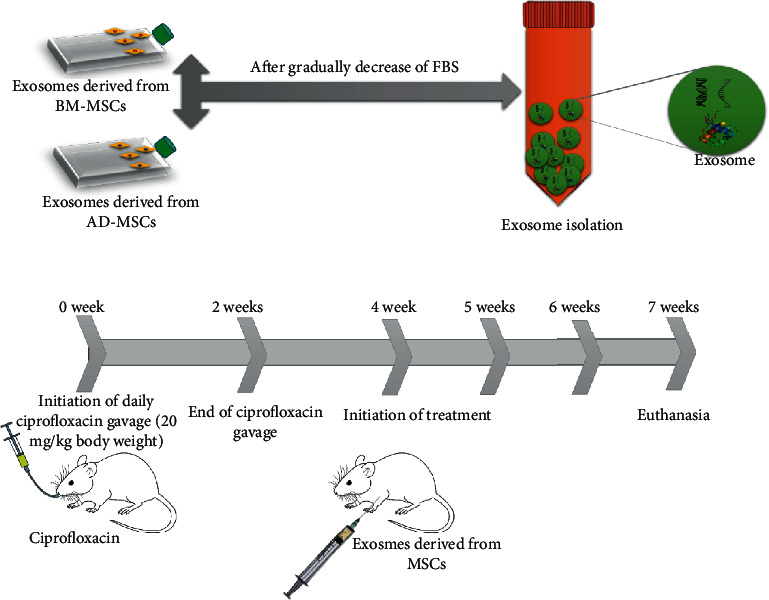
Schematic figure to explain the study design.

The procedure of the experiment is shown in the following schematic figure (Figure 1).

At last, to evaluate the changes in different groups, the expression levels of collagen I and II, sox9, and aggrecan genes using real-time PCR and histological analysis by a pathologist, as well as immunohistochemical studies, were performed. The complete description of the study steps is as follows.

### 2.2. Isolation and Culture of AD-MSCs

The adipose tissue samples were obtained from volunteer patients undergoing liposuction surgery who all signed the written informed consent. As described in our previous study [35], the isolation procedure was performed. Briefly, the adipose tissue samples were mechanically and then enzymatically digested using collagenase type I (1.5 mg/g) at 37°C for 45-60 min and cultured in Dulbecco's Modified Eagle's Medium (DMEM) supplemented with 1% penicillin-streptomycin and 10% fetal bovine serum (FBS) (Gibco, Grand Island, USA). The medium was changed every 3-4 days until the cells reach up to 80% confluence. For treatments, the cells were used in the 3rd passage.

### 2.3. Isolation and Culture of BM-MSCs

After obtaining written informed consent from the patients, bone marrow samples were aspirated and taken by an oncologist from preferably young people undergoing treatment for leukemia at Khorami Hospital in Qom province. Then, the aspirated samples were immediately transferred to the Stem Cell Laboratory of the Academic Center of Education, Culture, and Research (ACECR), Qom branch. To dilute the aspirated blood sample, an equal volume of PBS was added and then pipetted. The diluted blood was gently poured onto the equal volume of the Ficoll-Paque media (Lymphodex, innotrain, Germany) and then centrifuged at 2000 rpm for 25 minutes at 25°C. Mononuclear cells were separated from other layers, and about 4-5 mL of PBS was added and then centrifuged at 1300 rpm for 10 minutes at 25°C. The cell pellet was suspended in 1 mL of culture medium, and the suspension was added in a T25 flask containing 4 mL of DMEM medium (containing 10% FBS and 1% streptomycin/penicillin). Then, the cells were incubated at 37°C, 5% CO_2_, and 95% humidity. The medium was changed every 3-4 days, and when they reach 80% confluence, the cells were passaged. For treatments, the cells were used in the 3rd passage.

### 2.4. Flow Cytometry for Mesenchymal Stem Cell Markers

To confirm the isolated cells of bone marrow and adipose tissue as MSCs, the expression levels of positive cell surface markers (CD90, CD73, and CD105) and negative markers (CD34 and CD45) were determined. Flow cytometry was performed using an FC500 flow cytometer (Beckman Coulter, Fullerton, CA) and analyzed using the Beckman Coulter CXP software.

### 2.5. Preparation of Conditioned Medium

In the 3rd passage and 80% confluence of AD-MSCs and BM-MSCs, the culture medium was changed, and the new DMEM + FBS medium was replaced, and since then, with a gradual decrease in the amount of FBS, its level finally reached zero. The culture was then continued for another 48 hours. The collected conditioned medium was passed through a 0.2 filter. The collected medium could be kept at -80°C.

### 2.6. Isolating the Exosomes from Conditioned Medium

Separation of exosomes was performed using the Exocib exosome extraction kit according to the instructions of the manufacturer (Cib Biotech Co.). In summary, conditioned medium obtained from AD- and BM-derived MSCs was used to isolate exosomes. The sample was centrifuged at 3000 rpm for 10 minutes at 25°C to remove the excess particles. It was filtered through a 0.45 *μ*m syringe filter, and reagent A was added. After 5 min vortex and overnight incubation at 4°C, it was centrifuged, and reagent B was added. The isolated exosomes were kept at -80°C.

### 2.7. Characterization of Isolated Exosomes

#### 2.7.1. Flow Cytometry for the Surface Markers of Exosomes

Exosomes isolated from the conditioned medium of mesenchymal stem cells (from two sources of adipose tissue and bone marrow) were analyzed for CD63 and CD81 markers by flow cytometry. All the antibodies were used in flow cytometry experiments at the concentrations recommended by the manufacturers. Flow cytometry was performed using an FC500 flow cytometer (Beckman Coulter, Fullerton, CA) and analyzed using the Beckman Coulter CXP software.

#### 2.7.2. Exosome Identification by Transmission Electron Microscopy (TEM)

The isolated exosomes were fixed with 4% paraformaldehyde, applied onto formvar-coated carbon grids, and stained with 1% phosphotungstic acid at RT for 2 min. The morphological features of isolated exosomes were finally observed using an FEI Tecnai Spirit G2 transmission electron microscope (Thermo Fisher Scientific, Inc.).

#### 2.7.3. Bradford Assay for Detecting the Exosomes' Concentration

The Bradford protein assay is used to measure the concentration of total protein in a sample. To determine the number of isolated proteins, Bradford solution and standard diagram were studied using a Bicinchoninic Acid (BCA) protein assay kit (Sigma-Aldrich, Missouri, USA).

#### 2.7.4. Dynamic Light Scattering (DLS) of Exosomes

The size distribution of the exosomes was evaluated by DLS which illuminates the particles with a laser beam. All vesicles present in the beam will disseminate light, and the size distribution of these vesicles is assessed. The intensity fluctuations of the disseminated light will be measured, and a mathematical model derived from Brownian motion and light scattering theory will be applied. So, the size distribution of these vesicles is assessed [36]. For this aim, samples were diluted to 1 *μ*g/mL in PBS and 0.05% Tween-20, and the size of them was evaluated by DLS Zetasizer Nano ZS (Malvern Instruments, UK).

### 2.8. OA Induction in BALB/c Mice

This study was performed on 35 female BALB/c mice aged 3 weeks in the animal campus of Qom University of Medical Sciences, purchased from Royan Institute. Mice were randomly divided into 5 groups: normal (*n* = 5), OA (*n* = 5), AD-Exo (*n* = 10), BM-Exo (*n* = 10), and sham (*n* = 10). To induce OA, the mice received ciprofloxacin for 2 weeks at a dose of 20 mg/kg body weight by gastric gavage. On the 15th, 22nd, and 29th day of induction, mice in experimental groups of BM-Exo and AD-Exo were injected intra-articularly with 25 *μ*L BM-MSCs-derived exosomes (100 *μ*g/mL) and 25 *μ*L AD-MSCs-derived exosomes (100 *μ*g/mL), respectively, into the articular cartilage. Likewise, the sham group was injected with 25 *μ*L of PBS at the same time points. Normal and OA groups received no treatment. On day 36, the mice were euthanized for further evaluation.

### 2.9. Histology

The tibias of mice were fixed in 10% paraformaldehyde for 24 hours before being decalcified in 10% EDTA for 7 days at 37°C. Then, the samples experienced serial dehydration, and the tibial bones were embedded in paraffin, sectioned at a thickness of 5 m, and stained with hematoxylin and eosin (H&E) and safranin O/fast green. The severity of cartilage damage was histologically graded for each specimen for the medial tibial plateau by two blinded examiners using the Osteoarthritis Research Society International (OARSI) cartilage OA histopathology grading method.

### 2.10. Immunohistochemistry (IHC) Analysis

Collagen types I and II were stained using immunohistochemistry. The sections were deparaffinized, washed in PBS, antigen-retrieved, and blocked for 30 minutes with mouse IgG. Primary antibodies against mouse anti-collagen I (1 : 200; Novus Biologicals) and mouse anti-collagen II (1 : 200; Novus Biologicals) were incubated overnight on sections. The sections were then visualized using a biotinylated secondary antibody and streptavidin peroxidase solution.

### 2.11. Real-Time Polymerase Chain Reaction and Gene Expression Analysis

Total RNA was isolated from treated cells using “Gene All Kit (Gene All Biotechnology, Seoul, Korea) according to the manufacturer's instructions. RNA purity and quantity were assessed using Nanodrop 2000 spectrophotometer (Thermo Fisher Scientific, Wilmington, USA) at 260/280 nm. The reverse transcription was used to synthesize the first-strand cDNA using transcription Kit (Yekta tajhiz, Iran).” Quantitative real-time PCR assays were performed in triplicate to evaluate the expression of the selected genes. For normalizing gene expression levels, the glyceraldehyde-3-phosphate dehydrogenase (*GAPDH*) gene was used as an internal reference. The 2^-∆∆Ct^ method was used to calculate the fold change of mRNA expressions for target genes. Real-time PCR was carried out using RealQ Plus Master Mix Green (AMPLIQONIII) following the manufacturer's instructions. Briefly, a mixture comprised of 10 *μ*L SYBR green mix, 1 *μ*L of cDNA (250 ng), 1 *μ*L PCR forward primers and 1 *μ*L PCR reverse primer in 5 pmol *μ*L^−1^, and Millipore water to achieve a final volume of 20 *μ*L was made. The sequences of primers are presented in Table 1. The threshold cycle (CT) was determined manually for each run. Relative mRNA level was expressed as the relative fold change and calculated using the formula 2^−^ΔΔ^*CT*^ = 2^−^(Δ^*CT*(*Sample*)−^Δ^*CT*(*calibrator*)^), where each ∆CT = ∆CT Target–∆CT GAPDH.

### 2.12. Statistical Analysis

Experimental data were expressed as the mean ± standard error (SE) and compared by ANOVA. ANOVA using a multiple comparisons test was used to determine statistical significance, followed by the Tukey test. Statistical significance was set at *p* ≤ 0.05. All experiments were performed in triplicate for technical and biological repetitions.

## 3. Results

### 3.1. Characterization of AD-MSCs and BM-MSCs

**Figure 2:**
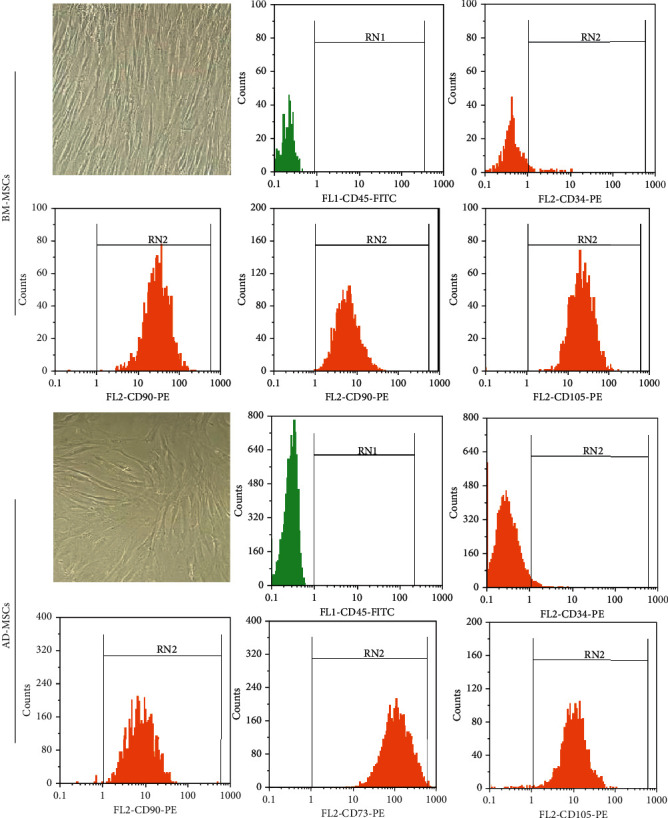
Characterization of MSCs derived from two sources of adipose and bone marrow tissues. The cell morphology and flow cytometric analyses of phenotypic markers of AD-MSCs and BM-MSCs were assessed.

Both the MSCs derived from adipose and bone marrow tissues exhibited a spindle-shaped, fibroblast-like morphology with clearly delineated cell margins. Also, flow cytometric analysis demonstrated that the majority of AD and BMMSCs were positive for CD73, CD90, and CD105 (>80%) but negative for CD45 and CD34 (≤3%) (Figure 2).

### 3.2. Characterization of AD-Exo and BM-Exo

**Figure 3:**
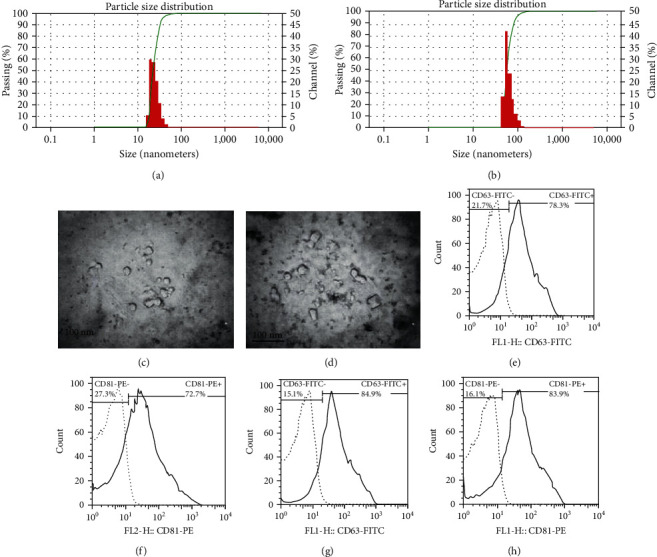
Characterization of isolated exosomes. Dynamic light scattering of AD-Exo (a) and BM-Exo (b) for measuring the mean size of these nanoparticles. Electron microscopy of AD-Exo (c) and BM-Exo (d). Flow cytometry of CD63 and CD81 markers showing positive results for AD-Exo (e, f) and BMMSC-Exo (g, h).

DLS analysis showed that the size of the majority of AD-Exo and BM-Exo was approximately 30–150 nm (Figures 3(a) and 3(b)). A cup-shaped or round-shaped morphology of AD-Exo and BM-Exo was revealed using transmission electron microscopy (Figures 3(c) and 3(d)). Also, flow cytometry of surface markers CD81 and CD63 was performed, and the result was positive for these two markers of both AD-Exo and BM-Exo (Figures 3(e)–3(h)).

### 3.3. Real-Time PCR Analysis

**Figure 4:**
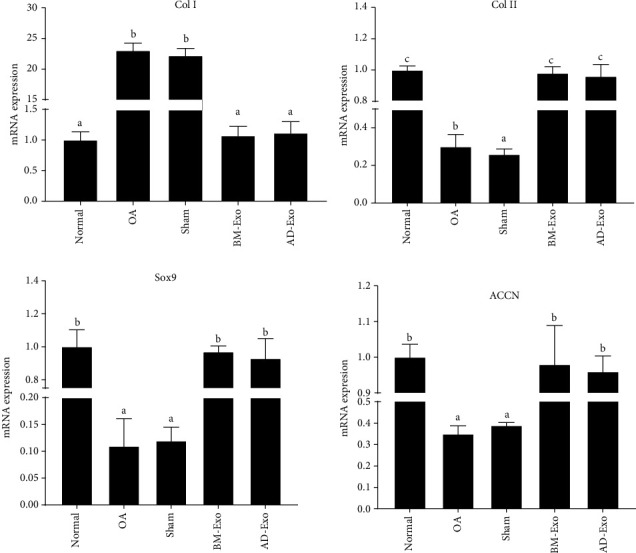
Comparison of collagen type I, Sox9, collagen type II, and aggrecan gene expression in cartilage tissue of different groups (*p* ≤ 0.05).

Examination of the real-time PCR results showed that the expression of collagen type I, Sox9, collagen type II, and aggrecan gene transcripts had changed after treating OA mouse models with AD and BM-Exo (Figure 4).

Based on the obtained data, the expression of Sox9, collagen type II, and aggrecan genes in the groups treated with AD-Exo and BM-Exo showed a significant increase in comparison to the OA group (*p* ≤ 0.05), while there was no significant difference between exosome-treated groups and normal mice (*p* > 0.05). On the other hand, the expression level of the collagen type I gene in the groups treated with AD-Exo and BM-Exo was significantly reduced compared to the OA group (*p* ≤ 0.05), but no significant difference was observed between the sham and the OA groups (*p* > 0.05).

### 3.4. Histologic Analysis

#### 3.4.1. Hematoxylin and Eosin (H&E) Staining

In the normal group, regular rows of chondroblasts and chondrocytes were observed in the cavities as isogenic groups. In addition, the boundary between each chondrocyte and the extracellular matrix was observed as a colored halo around the cell, indicating the presence of glycoproteins around the chondrocyte cavities. Normal bone tissue was observed below the area of articular cartilage in which cavities containing bone marrow tissue were observed coherently with a regular arrangement of osteocyte cells. Other joint structures such as the synovial membrane, lateral ligaments, and supporting muscles around the joint and joint capsule were observed normally. According to the osteoarthritis cartilage histopathology (OACH) assessment system [37], tissues in this group were considered as geade1. In the case of exosome-treated groups, the chondrogenesis process was significantly improved in the BM-Exo group; although in some areas, normal cartilage tissue was replaced by the collagen fibers as scar tissue. This group showed a better improvement compared to the AD-Exo group and was ranked in grade 2 whereas, in the AD-Exo group which was considered as grade 3, abnormal cartilage and bone fragments were still present in some areas.

**Figure 5:**
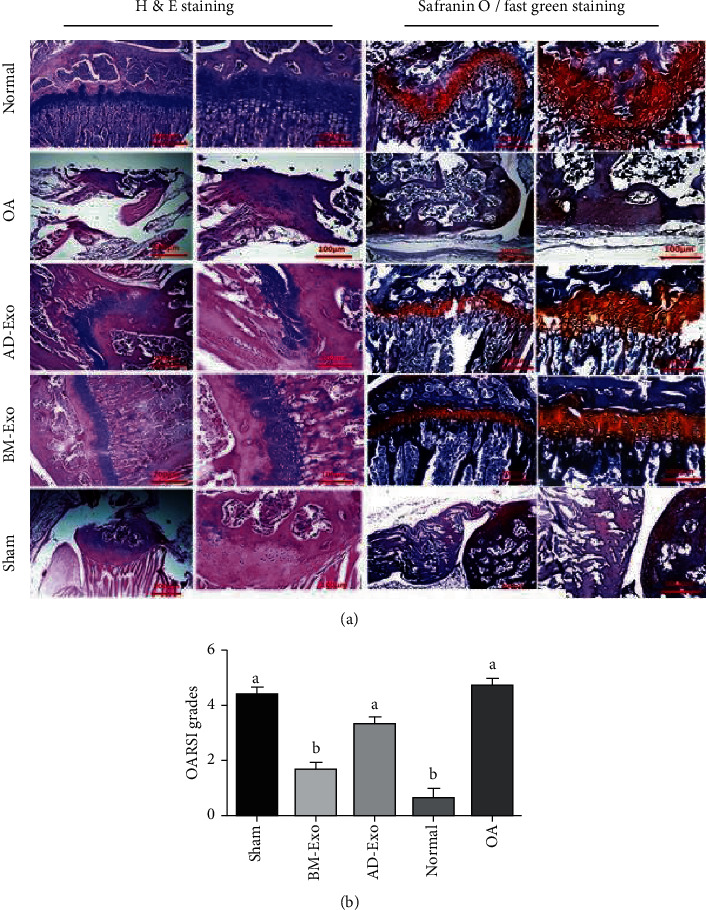
Histologic analysis of articular cartilage in normal, BM-Exo, AD-Exo, OA, and sham groups. (a) H&E staining and Safranin O/fast green staining images are presented in the left and right panels, respectively. (b) Statistical analysis of OARSI scores in the normal, BM-Exo, AD-Exo, OA, and sham groups (*p* ≤ 0.05). BM-Exo: exosomes secreted by bone marrow-derived mesenchymal stem cells; AD-Exo: exosomes secreted by adipose tissue-derived mesenchymal stem cells; H&E: hematoxylin and eosin; OA: osteoarthritis; OARSI: Osteoarthritis Research Society International.

In the two groups of sham and OA, a minimal amount of chondrogenesis with no significant difference was observed, and both were assigned in grade 5 (Figure 5).

#### 3.4.2. Safranin O/Fast Green Staining

The severity of cartilage damage in safranin O/fast green staining images (Figure 5) using the OARSI grading system [38] was assessed. OARSI scores in normal and BM-Exo groups were significantly lower than the OA group. On the other hand, the score of the BM-Exo group was significantly lower than the AD-Exo group, while no significant difference was observed between the BM-Exo and the normal groups (*p* ≤ 0.05) (Figure 5(b)).

### 3.5. Immunohistochemistry (IHC) Analysis

**Figure 6:**
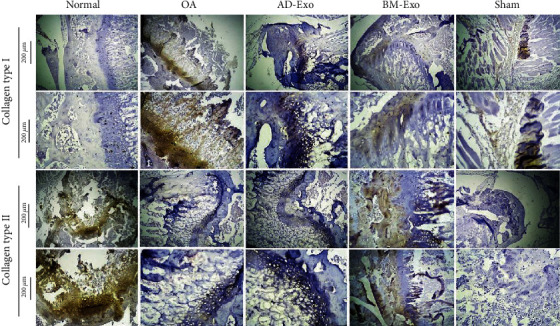
IHC analysis. Collagen I and II staining (brown coloration) in the normal, OA, AD-Exo, BM-Exo, and sham groups. OA: osteoarthritis; AD-Exo: exosomes secreted by adipose tissue-derived mesenchymal stem cells; BM-Exo: exosomes secreted by bone marrow-derived mesenchymal stem cells.

As it has shown in Figure 6, IHC analysis of articular cartilage showed that while collagen type I staining was not performed in the normal group and weak staining intensity was presented in AD-Exo and BM-Exo groups, but in the OA group, staining was done clearly and intensely. In addition, collagen type II staining was more severe in both exosome-treated and normal groups than the OA group. In fact, in the normal and BM-Exo groups, collagen II staining was seen in superficial and deep areas of cartilage, while in the AD-Exo group, collagen II was weakly observed in the superficial region (Figure 6).

Using the ImageJ software, the obtained images of IHC staining were quantified, and the expression of collagen I and collagen II proteins in different groups was statistically analyzed.

**Figure 7:**
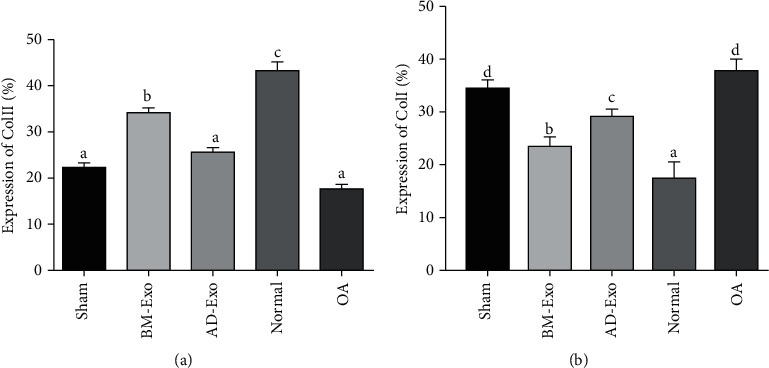
Comparison of quantified IHC results using the ImageJ software: (a) collagen I protein expression in different groups; (b) collagen II protein expression in different groups.

In the case of collagen II protein, the control group showed higher expression than the sham, groups treated with AD-Exo (*p* < 0.0001) and BM-Exo (*p* < 0.05). On the other hand, the group treated with BM-Exo showed significantly higher expression than the sham, OA (*p* < 0.0001), and AD-Exo (*p* < 0.05) groups, while increasing the collagen II expression was not significant in the AD-Exo group in comparison to the sham and OA groups (*p* > 0.05) (Figure 7(a)).

Besides, although the collagen type I protein expression was significantly lower in the control group than all the other groups (*p* < 0.05), its level in both exosome-treated groups was lower in comparison to the OA and sham groups (*p* < 0.05) (Figure 7(b)).

## 4. Discussion

Osteoarthritis disease is the most common challenge of aging, caused by dysregulation of the collagen type II and aggrecan level or the upregulation of collagen type I [39]. It is generally accepted that type II collagen is the main type of hyaline present in all cartilage tissues and is responsible for the stability and biological functions of healthy articular cartilage [40, 41].

MSCs as one of the most widely used stem cells due to their unique properties, including the ability to differentiate into several cell lineages and paracrine mechanisms that ultimately provide their potential for tissue regeneration, have attracted special attention in research and clinical studies for treating diseases [42]. However, therapeutic use of these cells has been restricted due to some problems associated with their possible immune safety profile such as tumorigenesis and subsequent innate and adaptive immune response [43]. As a result, alternative strategies seem to be needed to use effective biomolecules in disease treatment. One of these strategies is to use cell-free products secreted by stem cells [43]. A well-known candidate cell-free product that could play a role in tissue regeneration is the MSC-derived exosomes with small size and paracrine properties both suitable for regenerative medicine and tissue engineering [43, 44].

Based on the previous studies, the use of MSC-derived exosomes in OA chondrocytes caused a significant improvement in chondrocyte marker (type II collagen, aggrecan) expression [45–47], which are consistent with our study. In detail, Wang et al. showed that intra-articular injection of exosomes from embryonic MSCs restored the chondrocyte nature by enhancing collagen type II synthesis and reducing expression of a disintegrin and metalloproteinase with thrombospondin motifs 5 (ADAMTS5) in primary mouse chondrocytes treated with interleukin 1 beta (IL-1*β*) [47]. Furthermore, Cosenza et al. concluded that MSC-derived exosomes could support cartilage and bone from degradation in OA-like murine chondrocytes by providing a significant improvement in chondrocyte marker (collagen type II, aggrecan) expression [45]. Similarly, Qi and colleagues documented that using the conditioned medium from umbilical cord-derived mesenchymal stem cells could maintain the aggrecan and collagen II expression in nucleus pulposus- (NP-) like cells (NPCs) differentiated from MSCs exposed to high glucose [46]. In addition, Mao et al. reported that administration of miR-92a-3p-overexpressing exosomes from human BM-MSCs improves chondrogenesis and inhibits cartilage degradation by targeting Wnt family member 5A (WNT5A) [48]. Additionally, it was observed that the restorative effects of exosomes on osteochondral defects in rats [27] and rabbits [49] have also been shown to result in significant damage repair and hyaline cartilage formation containing high levels of collagen II. These effects induced an immune repair phenotype and increased cartilage metabolic activity in EV-treated injuries [27].

In the present study, for the first time, the effect of exosomes derived from BM-MSCs and AD-MSCs in the treatment of OA was investigated. The results showed that injection of AD-MSCs-exosome or BM-MSCs-exosome ameliorated OA in the ciprofloxacin-induced OA mouse model; however, BM-MSCs-exosome had a better therapeutic effect than AD-MSCs-exosome. Our outcomes are in agreement with part of the results reported by Cosenza et al. and Mao et al. that MSC-derived exosomes could remarkably increase chondrocyte marker expression and chondrogenesis [45, 48]. Also, it was verified that BM-MSCs may have a greater chondrogenic potential than AD-MSCs [34]. Due to the high chondrogenic potential of BM-MSCs than AD-MSCs, it is hypothesized that their resulting exosomes may also show a higher ability to differentiate into cartilage lineage than AD-Exo. Histological analysis showed that the regenerated cartilage in the BM-Exo group presented typical hyaline characteristics similar to normal cartilage which is in agreement with that reported by Zhu et al. that the regenerated cartilage in the exosome from induced pluripotent stem cells- (iPSCs-) derived mesenchymal stem cells (iMSCs) treated group has a normal hyaline feature similar to typical cartilage [7]. The cartilage repair process can be related to the ability of exosomes for tissue regeneration due to the presence of growth factors and cytokines.

Real-time PCR data of a primitive study showed a moderate increase in type II collagen mRNA in stage III compared with stage I OA. Also, it has been shown that the amount of type II collagen protein in the cartilaginous matrix decreases in the progression of osteoarthritis [41]. Similarly, the results of our study showed a reduced rate of type II collagen expression in the OA and sham groups compared to the normal group. However, in both exosome-treated groups, this difference was not statistically significant compared to the normal. In the case of collagen type I mRNA expression, also an increase was observed in the OA and sham groups compared to the normal group, while its expression level in BM-Exo and AD-Exo groups was similar to the control group. Our findings are consistent with some authors' reports which showed a decrease in type I collagen expression in vaginal fibroblasts from women with stress urinary incontinence after AD-exosome administration [50].

In our study, although the expression of collagen type II was lower in the BM-Exo and AD-Exo groups than the normal, the expression of this chondrogenic-related protein was significantly increased in both exosome-treated groups in comparison to the sham and OA, with higher expression in BM-Exo rather than AD-Exo.

## 5. Conclusion

This study indicates that both the exosomes from BM-MSCs and AD-MSCs present good opportunities as marvelous cell-based therapeutics for ameliorating osteoarthritis. Notably, exosomes from BM-MSCs in comparison with AD-MSCs could be considered as a better therapeutic option to improve osteoarthritis and exhibit potential as a disease-modifying osteoarthritis cell-free product.

## Figures and Tables

**Table 1:** Specific primers for target genes.

Name	Primer sequence (5′-3′)	Product size	Annealing TM (°C)
Collagen I	F: CCAGCCGCAAAGAGTCTAC R: TCAGGGATGTCTTCTTGGC	119	57
Sox9	F: TGCCTGGAAACTTCTGTGG R: GGAGGGAAAACAGAGAACG	112	57
Collagen II	F: TAACACCCCAGGAGGATGC R: AGGGAGATGGGACACTTGC	240	59
Aggrecan	F: GAAGTTCTTGGAGGAGCGAG R: GGGATGCTCACGCTCAGT	142	60
GAPDH	F: AAGGTCATCCCAGAGCTGAA R: CTGCTTCACCACCTTCTTGA	222	59

## Data Availability

The data used to support the findings of this study are available from the corresponding author upon request.
